# Supplementary material for: Interaction of amisulpride with GLUT1 at the blood-brain barrier. Relevance to Alzheimer’s disease
Source: PLoS One. 2023 Oct 24;18(10):e0286278. doi: 10.1371/journal.pone.0286278 (PMC10597500; doi:10.1371/journal.pone.0286278)
Supplement: S1 Raw images — (PDF) [file pone.0286278.s002.pdf]

A

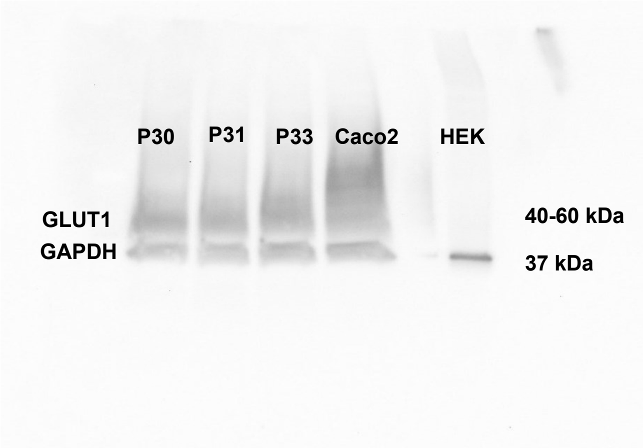

Fig 4A. GLUT1 expression in hCMEC/D3 cells. Three passages of hCMEC/D3 cells (P30, P31 and P33) (30 µg of protein per well) were tested for GLUT1 (40-60 kDa) expression. The figure is an example membrane of three technical repeats. Caco-2 cell lysate was used as a positive control; HEK-293 cell lysate was used as a negative control. GAPDH (37 kDa) was used as a loading control. Antibodies used: anti-GLUT1 antibody – 1:100 000, #ab115730; anti-GAPDH antibody – 1:2500, #ab9485, Abcam; secondary anti-rabbit IgG, HRP-linked antibody – 1:2000, #7074, Cell Signalling Technology.

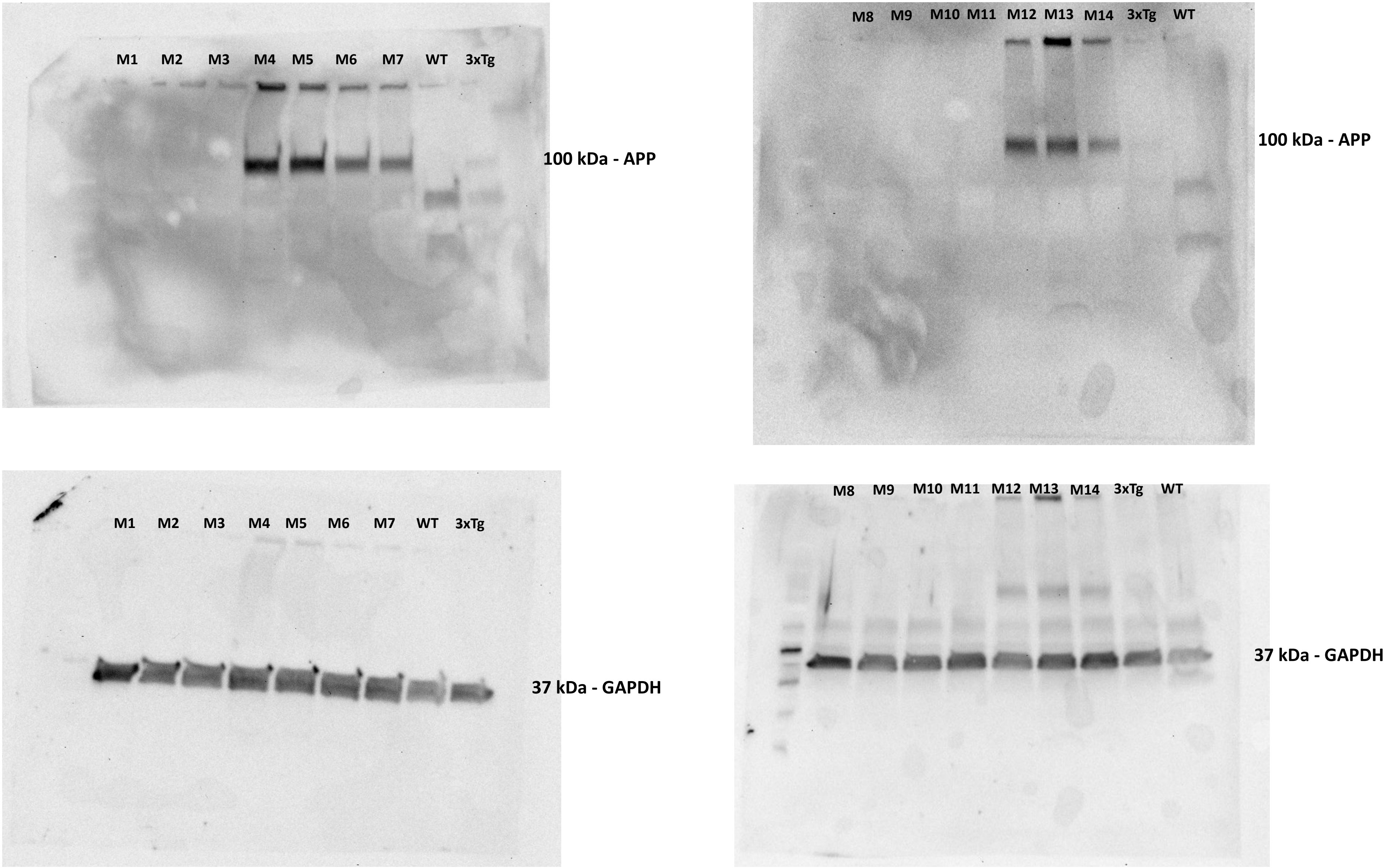

Supplementary Figure 4: Capillary lysates (40 µg per well were loaded) from WT and 5xFAD mice stained with anti-beta Amyloid 1-16 antibody (# 803004, Biolegend, RRID: AB_2715854, detecting APP and Aβ) (n=3 WT and n=4 5xFAD, one membrane M1-7; n=4 WT and n=3 5xFAD, second membrane, M8-14). GAPDH (37 kDa) was used as a loading control. For the anti-beta amyloid antibody, a secondary anti-mouse antibody was used – 1:3000 (#ab6728, Abcam, UK, RRID:AB_955440). For GAPDH, a secondary anti-rabbit IgG, HRP-linked antibody was used – 1:1000 (#7074, Cell Signalling Technology). Brain capillary lysates from a known WT and 3xTg mice were used as a negative and a positive control, respectively.

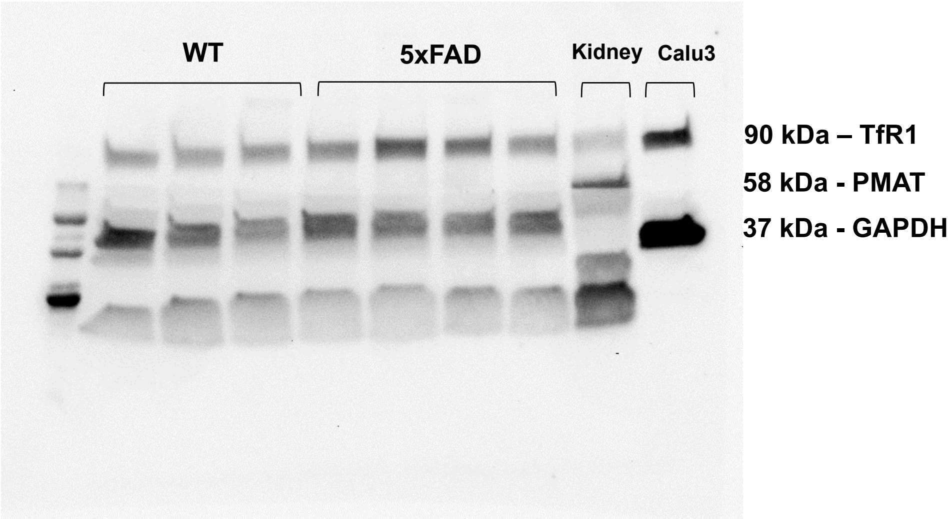

Supplementary Figure 5: Brain capillary lysates (40 µg per well were loaded) from C57BL/6 and 5xFAD mice were tested for TfR1 (90 kDa), PMAT (58 kDa) expression, (n=6 WT mice, n=7 5xFAD mice across two membranes, three technical repeats of each, one membrane is presented). GAPDH (37 kDa) was used as a loading control. Mouse kidney lysate was the positive control for PMAT. Calu3 was the negative control for PMAT. Antibodies used: anti-TfR1 antibody – 1:1000, #13-6800, Thermo Fisher; anti-PMAT antibody – 1:600, #bs-4176R, Bios; anti-GAPDH antibody – 1:2500, #ab9485, Abcam; secondary anti-rabbit IgG, HRP-linked antibody – 1:1000, #7074, Cell Signalling Technology for PMAT and GAPDH; secondary anti-mouse antibody – 1:3000 (#ab6728, Abcam, UK, RRID:AB_955440) for TfR1.

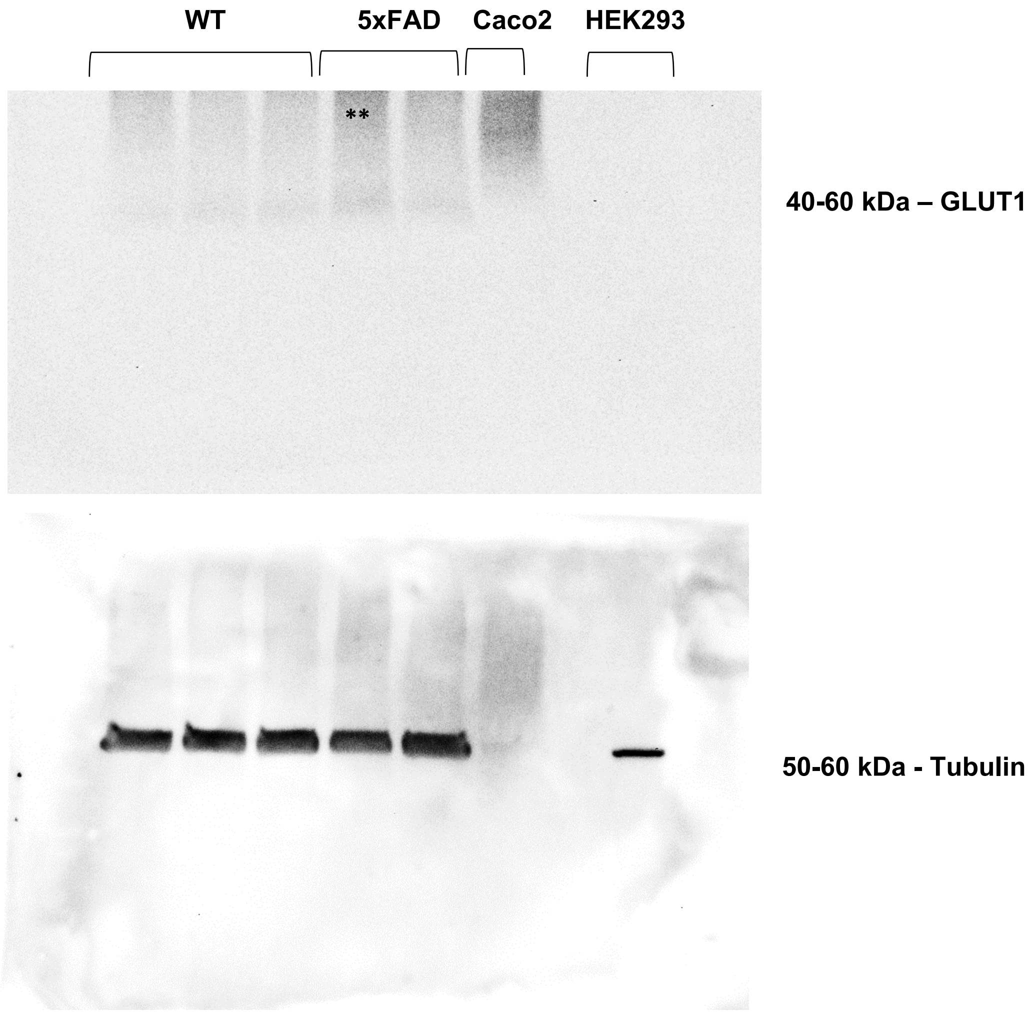

Supplementary Figure 6: Brain capillary lysates (20 µg per well were loaded) from C57BL/6 and 5xFAD mice were tested for GLUT1 (40-60 kDa), (n=5 WT mice, n=4 5xFAD mice across two membranes, three technical repeats of each, except ** samples – which have two repeats, and * – which have one repeat, one membrane is shown). Tubulin (50-60 kDa) was used as a loading control, Caco2 and HEK293 (#ab7902, Abcam) whole cell lysates were used as a positive and negative control, respectively. Antibodies used: anti-GLUT1 antibody – 1:100 000, #ab115730, Abcam; anti-Tubulin clone DM1A antibody – 1:4500, #05-829, Millipore;, Cell Signalling Technology, secondary anti-mouse antibody IgG, HRP linked antibody – 1:2000, #ab6728, Abcam.

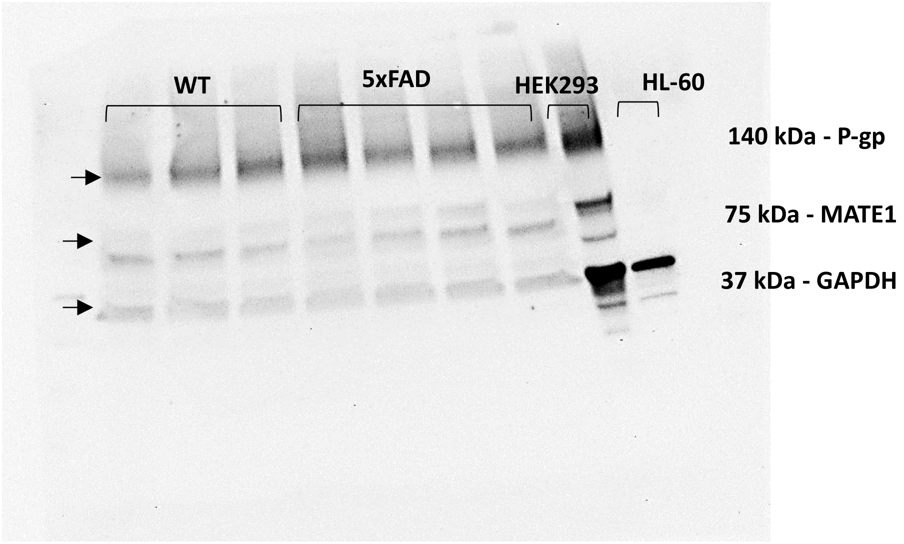

Supplementary Figure 7: Brain capillary lysates (40 µg per well were loaded) from WT and 5xFAD mice were tested for P-gp (141 kDa), and MATE1 (75 kDa) expression, (n=6 WT and n=7 5xFAD mice per group using three membranes, one membrane is presented). GAPDH (37 kDa) was used as a loading control. Antibodies used: anti-P-gp antibody – 1:1000, #ab170904, Abcam; anti-MATE1 – 1:1000, #ANT-131, Alomone Labs; anti-GAPDH antibody – 1:2500, #ab9485, Abcam; secondary anti-rabbit IgG, HRP-linked antibody – 1:1000, #7074, Cell Signalling Technology. Kidney lysates from a C57BL6 mouse and HEK293 whole cell lysate (#ab7902, Abcam, UK) were used as positive controls for P-gp and MATE1, HL-60 whole cell lysate (#ab7914, Abcam, UK) was used as a negative control for P-gp and MATE1.

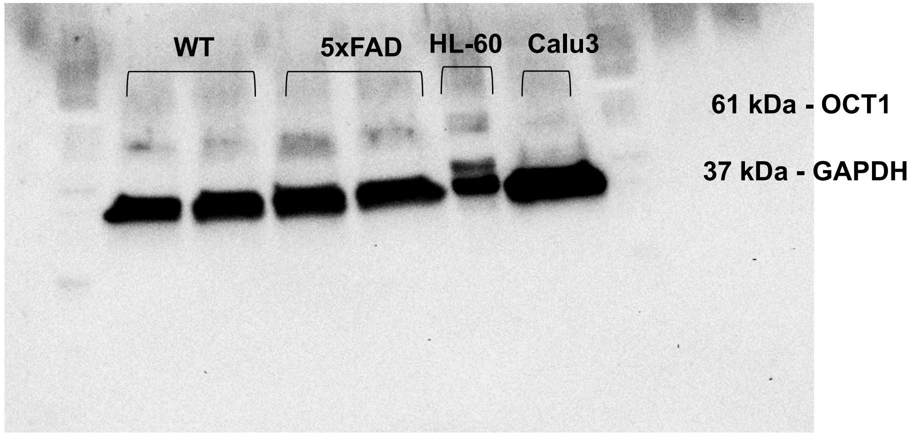

Supplementary Figure 8: Mouse brain capillary lysates (40 µg per well were loaded) from WT and 5xFAD mice were tested for OCT1 (61 kDa) expression, (n=5 WT and n=6 5xFAD mice per group using three membranes, one membrane is shown). GAPDH (37 kDa) was used as a loading control. Antibodies used: anti-OCT1 antibody – 1:667, #ab55916, Abcam; anti-GAPDH antibody – 1:2500, #ab9485, Abcam; secondary anti-rabbit IgG, HRP-linked antibody – 1:1000, #7074, Cell Signalling Technology. HL-60 cell line lysates (#ab7914, Abcam, UK) and Calu3 cell lysates were respectively used as a positive and negative control for OCT1.

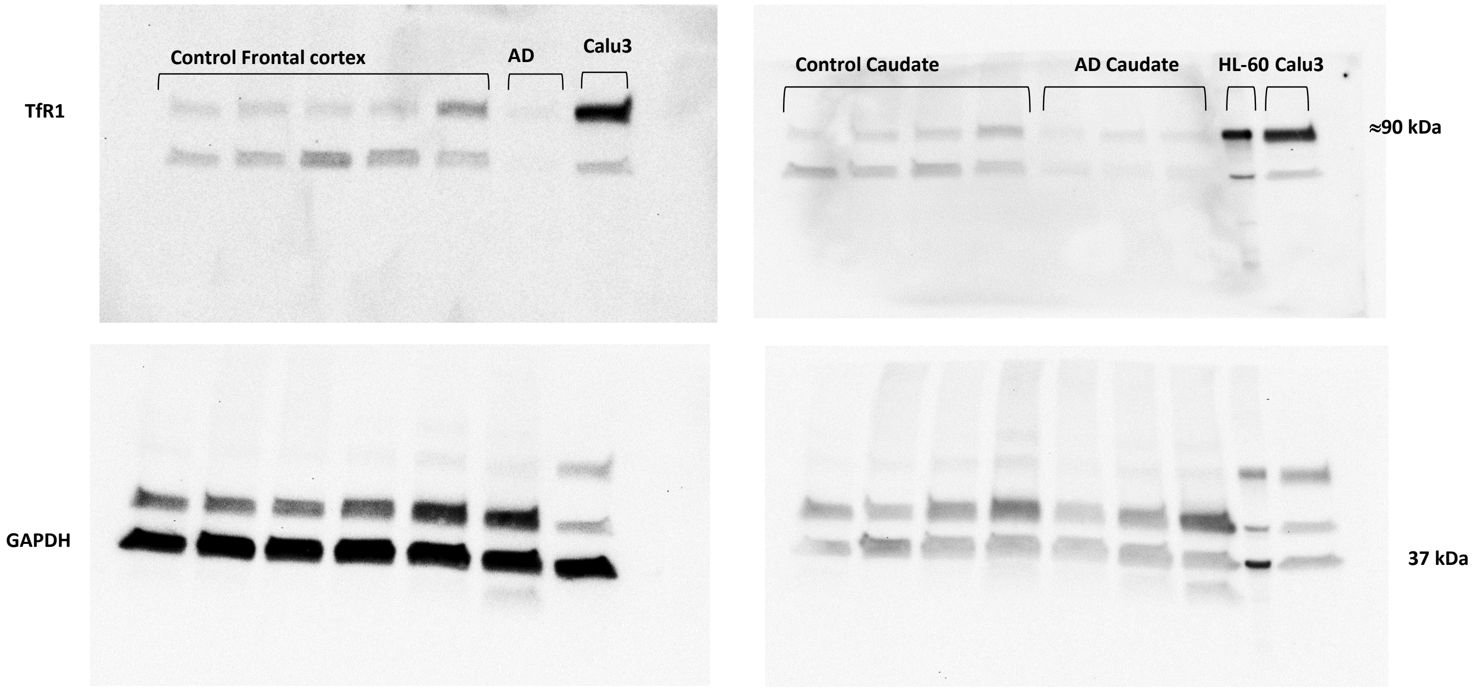

Supplementary Figure 11: Frontal cortex capillary lysates (25 µg per well were loaded) from control and AD cases were tested for TfR1 (90 kDa) (n=9 Control, n=9 AD cases across 3 membranes, three technical repeats of each). Caudate capillary lysates (25 µg per well were loaded) from control and AD cases were tested for TfR1 (90 kDa), (n=7 Control, n=8 AD cases across three membranes, three technical repeats of each, except for one control sample). GAPDH (37 kDa) was used as a loading control. Antibodies used: anti-TfR1 antibody – 1:1000, #13-6800, Thermo Fisher, secondary anti-mouse IgG H&L HRP-linked antibody – 1:3000, #ab6728, Abcam; anti-GAPDH antibody – 1:2500, #ab9485, secondary anti-rabbit HRP-linked antibody – 1:2000, #7074, Cell Signalling Technology.

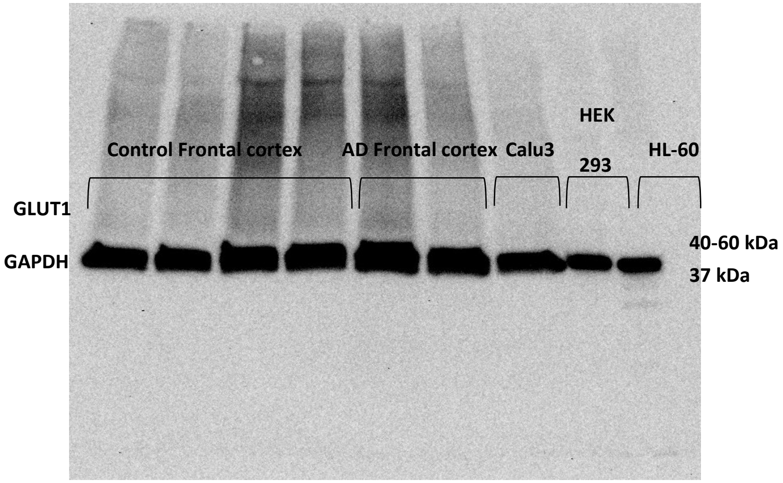

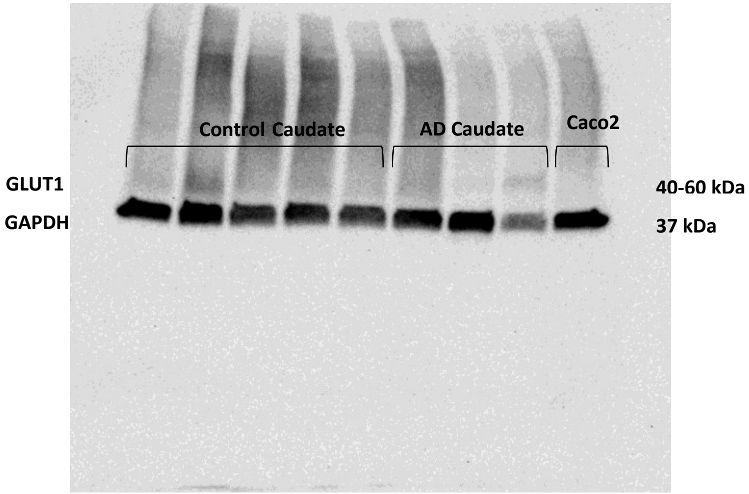

Supplementary Figure 12: Frontal cortex capillary lysates (20 µg per well were loaded) from control and AD cases were tested for GLUT1 (40-60 kDa), (n=9 Control, n=13 AD cases across four membranes, three technical repeats of each, except for one AD sample). Caudate capillary lysates (20 µg per well were loaded) from control and AD cases were tested for GLUT1 (40-60 kDa), (n=9 Control, n=9 AD cases across three membranes, three technical repeats of each). One membrane is shown. GAPDH (37 kDa) was used as a loading control, Caco2 lysates were used as a positive control, HEK293 cell lysates and human promyelocytic leukaemia cell line (HL-60) were used as negative controls for GLUT1. Antibodies used: anti-GLUT1 antibody – 1:100 000, #ab115730, Abcam; anti-GAPDH antibody – 1:2500, #ab9485, Secondary anti-rabbit HRP-linked antibody – 1:2000, #7074, Cell Signalling Technology.

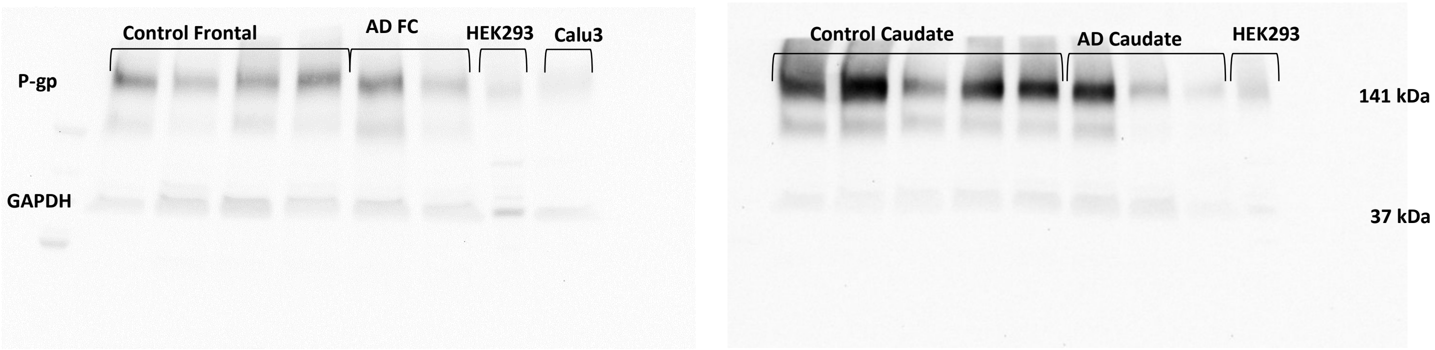

Supplementary Figure 13: Frontal cortex capillary lysates (20 µg per well were loaded) from Control and AD cases were tested for P-gp (141 kDa), (n=9 control, n=9 AD cases across three membranes, three technical repeats of each). Caudate capillary lysates (20 µg per well were loaded) from Control and AD cases were tested for P-gp (141 kDa), (n=9 Control, n=7 AD cases across three membranes, three technical repeats of each). GAPDH (37 kDa) was used as a loading control. HEK293 cell lysate was used as a positive control for P-gp. Antibodies used: anti-P-gp antibody – 1:1000, #ab170904, Abcam, Secondary anti-rabbit HRP-linked antibody – 1:2000, #7074, Cell Signalling Technology; anti-GAPDH antibody – 1:2500, #ab9485, Secondary anti-rabbit HRP-linked antibody – 1:2000, #7074, Cell Signalling Technology.
