# Supplementary material for: Interaction of amisulpride with GLUT1 at the blood-brain barrier. Relevance to Alzheimer’s disease
Source: PLoS One. 2023 Oct 24;18(10):e0286278. doi: 10.1371/journal.pone.0286278 (PMC10597500; doi:10.1371/journal.pone.0286278)
Supplement: Attachment — Submitted filename: Response to Reviewersv2.docx [file pone.0286278.s003.docx]

We would firstly like to take this opportunity to thank the reviewers for their constructive comments.

**Q1: The reviewers mentioned that the suitability of the model systems should be justified.**

**IN RESPONSE:**

We have now provided further justification of the methods used in the manuscript.

In this manuscript multiple approaches were used to test the hypotheses that amisulpride interacts with GLUT1 at the BBB, and that the expression of BBB transporters and the transport of amisulpride and glucose into the brain will be affected by AD.

**As stated on line 136:**

‘We utilised a combination of literature review, physicochemical properties analysis, molecular docking approaches, cell culture BBB studies, studies in wild type mice and in an animal model of AD and assessment of tissue from human cases with and without AD. An overview of the methods deployed are presented in Supplementary Fig 1, S1 File’.

**Literature review**

**As stated on line 155:**

‘Three different groups of molecules, which interacted with GLUT, were identified by literature review. These groups were GLUT1 substrates, GLUT1 inhibitors and GLUT-interacting antipsychotics’.

In fact we were able to identify:

**As stated on line 652:**

9 GLUT1 substrates, 33 GLUT1 inhibitors, and 11 GLUT-interacting antipsychotics

**Physicochemical properties analysis**

This was followed by physicochemical characterization of the three groups using specialist chemical property databases.

**We have now added more information to line 194:**

‘The physicochemical characteristics of the three groups were compared to those obtained for amisulpride to examine any overlap and identify differences’.

This information provided the basis for the *in silico*, *in vitro* and *in vivo* experimental design described below.

***In silico* approaches**

*In silico* molecular docking studies allowed us to explore the specific molecular interactions of amisulpride, alpha-D-glucose, beta-D-glucose, and sucrose with GLUT1.

**We have now further justified the *in silico* models by adding the following sentence to the manuscript: Line 200:**

‘We have already studied the interaction of amisulpride with several transporters (e.g. MATE1, PMAT, organic cation transporter 1 (OCT1) and P-gp) using molecular docking (Sekhar et al., 2019).’

**Cell culture models**

**We have now further justified the cell culture models by adding the following information to the manuscript:**

**Line 221:**

The human cerebral microvessel endothelial cells/D3 (hCMEC/D3) are an immortalised human adult brain endothelial cell line that stably maintains a BBB phenotype.

We have also added the following sentence Line 223:

‘It is known to express ABC and SLC transporters including P-gp, OCT1, OCT2, OCT3, organic cation transporters novel 1 (OCTN1), OCTN2, PMAT, MATE1 and MATE2 (Sekhar et al., 2017; 2019)’.

We have also added the following information to line 227:

This *in vitro* method allowed us to: (1) study human brain endothelium (2) confirm that GLUT1 was expressed in these cells (3) confirm presence of a glucose transporter (4) study the interaction of amisulpride with a glucose transporter (5) minimize time-consuming animal studies in line with the framework of the replacement, reduction and refinement (3Rs) principles.

**Animal model of AD and WT**

**We have added the following information to line 369:**

Comparing data obtained from this mouse model of AD to that obtained from WT mice allowed us to explore the effect of disease on neurovascular structures, transporter expression in brain capillaries and on drug delivery across the BBB.

**Human tissue**

We have added the following sentence to the manuscript to further justify use of human tissue: Line 1009:

‘We also explored the expression of SLC and ABC transporters in the same brain capillary sample and assessed regional differences.

Our responses to other comments are listed below.

**Q2. Reviewer #1: Reviewed manuscript contains very important informations for doctors, researchers and for patients with AD. Therefore, I think, this subject should be continued. Short suggestion: For names of genes should be used italic as a font. Therefore, write please (LINE 105) name of gene (SLC2A1) according to presented suggestion.**

**Q2: RESPONSE:** We have reviewed each sentence which includes an SLC code to determine if we were referring to the protein or the gene.

We specified a specific SLC code three times (Lines 108, 109 and 113).

On line 108 we specified SLC47A1 and on line 109 we specified SLC29A4.

The reference we cited was:

16. Sekhar GN, Fleckney AL, Boyanova ST, Rupawala H, Lo R, Wang H, et al. Region-specific blood–brain barrier transporter changes leads to increased sensitivity to amisulpride in Alzheimer’s disease. Fluids Barriers CNS. 2019 Dec 17;16(1):38.

We re-examined the reference and, in both instances, we were referring to the protein code so the SLCs should not be italized. We have now confirmed this point in these sentences by addition of the word: protein.

On line 113, we specified SLC2A1. We re-examined the three papers cited. References 18-20.

18. Horwood N, Davies DC. Immunolabelling of hippocampal microvessel glucose transporter protein is reduced in Alzheimer’s disease. Virchows Arch. 1994;425(1):69–72. PROTEIN

19.	Mooradian AD, Chung HC, Shah GN. GLUT-1 expression in the cerebra of patients with Alzheimer’s disease. Neurobiol Aging. 18(5):469–74. PROTEIN

20.	Kalaria RN, Harik SI. Reduced glucose transporter at the blood-brain barrier and in cerebral cortex in Alzheimer disease. J Neurochem. 1989 Oct;53(4):1083–8.

In all cases the changes that were observed were because of protein changes or changes in function. So the SLC code should not be italized.

**Q3. Reviewer #2: This manuscript reports a gravely flawed analysis of the interactions of an antipsychotic drug (amisulpride) with aspects of cerebrovascular transport, including attempts at relevance to Alzheimer’s disease. The approach included literature review to identify and characterize confirmed and proposed antipsychotics that interact with glucose transporter 1 (GLUT1), in-silico modeling of amisulpride with GLUT1, in-vitro tests of glucose uptake in a transformed endothelial cell line, and a in-vivo perfusion assay of amisulpride uptake into the brain of a mouse model of amyloid accumulation. Levels of some transport enzymes (including GLUT1) in mouse and human brain were also assayed, and the ultrastructural integrity of cerebrovascular structure was evaluated in an Alzheimer’s case. Despite the lack of physicochemical data to this effect, the authors conclude that amisulpride may alter glucose uptake by GLUT1 and that this necessarily applies to other antipsychotic drugs. They also conclude that levels of GLUT1 are not altered in AD or a mouse model of amyloid accumulation and that P-gp levels are lower in AD brains but only in the caudate.**

**Q3 RESPONSE:** The reviewers summary of the manuscript is incorrect.

Firstly, we are not saying that amisulpride may alter glucose uptake by GLUT1 and that this applies to other antipsychotic drugs.

We have *in silico* evidence that amisulpride interacts with GLUT1 and other groups have evidence that other antipsychotic drugs interact with glucose transporters including GLUT1. In fact, 11 antipsychotics were identified which interact with glucose transporters (see Supplementary Table 5, S1 File).

So there is evidence from other sources that would suggest amisulpride has potential to interact with GLUT.

Secondly, we also did not assess ‘enzymes’ we assessed the expression of ‘transporters and receptors’.

Thirdly, the physicochemical characterization data does not allow us to rule out that amisulpride interacts with GLUT.

Regarding the physicochemical characteristics:

-The +1 charge of amisulpride is similar to the other antipsychotics that interact with the glucose transporters.

-In addition, we identified one GLUT1 substrate that has a +1 charge so there is evidence that other types of molecules that interact with GLUT 1 have a similar charge.

-The MW of amisulpride suggested it would more likely be an inhibitor of GLUT1 transport than a GLUT1 substrate due to its similarity to other GLUT1 inhibitors.

**Q4: The perfusion approach used to assess amisulpride uptake is unusual and likely inappropriate. Similar assays of GLUT1 transport of glucose or its analogs is performed on awake, unanesthetized animals because anesthetics interfere with uptake and/or metabolism. Here, mice were anesthetized with medetomidine and ketamine. Ketamine has been shown to have direct effects on GLUT1 (PMID: 10927023). Medetomidine is an alpha-adrenergic R2 agonist, and these receptors have dramatic effects on glucose regulation. Whether or not there is a more proximal effect of medetomidine on GLUT1, the increase in blood glucose levels that results from alpha-adrenergic R2 agonism would certainly make an appreciable impact on GLUT1 function. Incidentally, it is never stated where was the perfusion was introduced; one assumes it was performed transcardially, as was the perfusion for fixation.**

**Q4: RESPONSE:**

*Anaesthetic use:* The reviewer is concerned that the anaesthetics will alter the glucose concentration of the plasma. However, the plasma used in the perfusion assay is artificial and as a result the concentration of glucose in the plasma is stable throughout the experiment.

*Specify route of perfusion:* The reviewer mentioned we had not specified the route of perfusion*.*

On line 450 we state that the: ‘The mouse brain was *in situ* perfused via the heart with artificial plasma.’

However, we have now included an additional sentence to explain this in more detail:

On line 499: ‘The artificial plasma was perfused through the left ventricle of the heart. The right atria was sectioned to allow outflow of the inflowing artificial plasma’.

**Q5: The hCMEC/D3 cell line is not a valid model for glucose uptake by cerebrovascular endothelium. These cells have been immortalized by viral transduction of telomerase (hTERT) and SV40 large T antigen. This has almost certainly altered their glucose metabolism and transport (PMID: 2998464, PMID: 1316810, PMID: 11390181). Among other alterations, hCMEC/D3 cells express considerable amounts of GLUT3 and SGLT1, which are not present in normal endothelial cells, in mouse at least. If they are significantly expressed normal human cerebrovascular endothelium, this would behoove the authors to include these proteins in their analysis of AD versus control**.

**Q5: RESPONSE:**

5a) We would like to highlight the following paragraph to the reviewers:

Please note the bolded sentences.

**Lines 1103-onwards.**

‘We confirmed the expression of GLUT1 in the hCMEC/D3 cells with WBs. These results are in line with previous studies. GLUT1 protein expression has been detected in hCMEC/D3 cells and in brain microvascular endothelial cells derived from healthy patient-derived induced pluripotent stem cells (61,62). Other glucose transporters’ mRNA has also been detected in the hCMEC/D3 cells, namely GLUT3 and sodium-dependent glucose transporter 1 (SGLT1) (62,63). Protein expression of GLUT3 and GLUT4, but not SGLT1, has also been reported in hCMEC/D3 cells (61). **Nevertheless, there is consensus that GLUT1 is the most highly expressed transporter in the hCMEC/D3 cells and the BBB** (64).’

**5b) Line 1106: We have now added that:**

-‘Importantly, GLUT-1 protein expression in hCMEC/D3 cells was found by quantitative proteomic analysis to be similar to that found in human brain microvessels (Ohtsuki et al., 2013)’**.**

**Line 1113: We have now added that:**

**-**Hexose transport has previously been examined in hCMEC/D3 cells (Meireles et al., 2013).

**5c) We have also been more general when describing the *in vitro* hCMEC/D3 accumulation assay.** So we do not specify GLUT1 transport exactly we soften the statements to say glucose transport or glucose transporters e.g. Lines 282, 773,774, 784, 804, 814, 822, 831, 1001, 1002, 1114, 1123, 1126, 1128, 1280 and 1282.

This clarifies that the experimental design allows assessment of glucose transport by any glucose transporter expressed in the hCMEC/D3 and not just glucose transport by GLUT1. We have specifically stated this now on Lines 1114-1116.

**Q6: TEM analysis of vascular morphology was performed on only one human sample (AD).**

**Q6 RESPONSE:** Data from human tissue samples are rare and it is not a simple process to study these precious samples. In particular, samples for TEM must have a very brief post-mortem delay (i.e. up to 22 h) and be processed for TEM in a specific manner.

We were unable to obtain any further samples which had a PMD less than 22 hours in the time frame of this study. However, we considered that the importance and rarity of the images from the one donor that we did have, merited inclusion in this primary research paper and would be of great interest to PLOS One readers.

**Q7: Why was glucose uptake represented in units of volume? How can this make sense? It seems to assume that cells import glucose by pinocytosis of the buffer or medium surrounding them.**

**Q7: RESPONSE:**

We have now more clearly explained the expression of results section:

Line 339-

The radioactivity (dpm) accumulated in the cells per mg of protein was expressed as a function of the radioactivity in a μl of the initial accumulation buffer to calculate the volume of distribution (V_d_; ml/mg of cellular protein) of the [^14^C]D-glucose or [^3^H]mannitol as shown in the equation below.

$$Vd=\frac{dpm per mg ofprotein}{\begin{aligned} dpm per \mu l of accumulation buffer \end{aligned}}$$

The V_d_ for [^14^C]D-glucose can be corrected with the V_d_ for [^3^H]mannitol (marker for non-specific binding) at each time point. Values were expressed as a mean±SEM. The [^3^H]mannitol values can also be used to confirm membrane integrity of each monolayer. [^3^H]mannitol is a well-established marker of BBB integrity (Kadry et al., 2020). This method allows us to compare the accumulation of a radiolabelled test molecule into human brain endothelial cells after a set of different experimental conditions such as self-inhibition (ie. plus unlabelled glucose) or cross-inhibition (i.e. plus unlabelled amisulpride) studies. In this way we can explore the means of cell entry and even cell exit of the test molecules. Movement across the cell membranes may be by passive diffusion, transporter mediated or indeed involve vesicles which may be receptor mediated.

**Q8 Suppl. Fig. 4 is troubling. Many of the 5xFAD mice appear to lack APP, as does the “positive control” 3xTg. Some of the samples are even inconsistent between the two gels: M4 and M5 have no APP in the first membrane but robust levels in the second, and M3 is the opposite. It is very difficult to understand how the authors can claim “WB analysis validated the 5xFAD mouse model for our study.” [Lines 1126–1127]**

**Q8 RESPONSE:**

Thank you for noticing this.

We have replaced the supplementary Figure 4 images with higher resolution images. In these images you can see the APP in the 3xTG lane. It is noted it is faint.

We have also renumbered the lanes and the mice. There were two cohorts of mice and the mice were given the same identifier in the initial Supplementary Figure 4, hence the confusion. We have corrected this.

**Q9: It is inappropriate to heat samples to 95 deg-C before western-blot analysis of** **membrane transport enzymes. Such high heat causes stable aggregation of GLUT1 and P-gp, retarding entry of a large fraction of these proteins into polyacrylamide gels. Suppl. Figs. 6 and 12 makes it clear that GLUT1 was not successfully detected or quantified in this study, rendering the “data” in Fig. 10 and Suppl. Fig. 9 meaningless.**

**Q9 RESPONSE:**

The antibody manufacturer advises against boiling the samples (<https://www.abcam.com/products/primary-antibodies/glucose-transporter-glut1-antibody-epr3915-ab115730.html#lb>). Yet due to sensitivity issues with a previous GLUT1 antibody used, we had tested in the lab, heating (95°C or 100°C), and not heating (room temperature), and the heating condition was picked due to the stronger signal obtained. The heat-related issue has now been noted in the methods (Lines 268-274) and discussion (Line 1256).

**Lines 268-274:**

**‘**Optimization assays were performed to compare samples heated at 95°C for 5 minutes to samples left at room temperature. The heated samples gave a stronger signal. Heating samples has previously been utilized for the Recombinant Anti-Glucose Transporter GLUT1 antibody and an anti-Pgp antibody (Ji et al., 2023; Morimoto et al., 2022).

**It has also been noted in the discussion.**

**Line 1256:** This could be related to the effects of the thermal processing of the samples, which could have prevented some of the protein from entering the gel and/or production of multimers which can be mistaken for background.

**Q10: Suppl. Table 2 indicates 9 and 14 samples were available for controls and AD, respectively. But not all of these were analyzed for every protein target. How did the authors select those which were omitted? Was there randomization?**

**Q10 RESPONSE:**

This was due to the availability of certain processed samples in different time frames. They were not deliberately omitted it was just availability of processed samples.

We have now provided more details of the specific cases used for each protein in the Supplementary Table 7, S1 File.

We have mentioned this on Line 981-983: Details of the specific samples used in the Western blot studies are tabulated in Supplementary Table 7, S1 File.

**Q11: A statistician should be consulted regarding the use of t tests for analyses that include several brain regions or other variables that result in multiple measurements (e.g., Figs. 7 and 10). Most statisticians would caution against this due to the introduction of risk for Type-1 error.**

**Q11 RESPONSE:
Interpretations/conclusions**

**FIGURE 7**

-Fig 7A: Normal two-way ANOVA or repeated measures requires the same n of data points for each group, so when some data points are missing, GraphPad Prism v9 statistical analysis software performs a mixed-effects analysis. In the case of the mouse perfusion data, the data was arranged for repeated measures analysis (since we have multiple samples from the same mouse) but was analysed with mixed-effects analysis due to the missing values.

-We have explained more clearly the types of statistical tests utilized in the Figure 7.

We now state in the text:

-Lines 522-529: The difference between WT and 5xFAD mice in the [^3^H]amisulpride uptake in the different brain regions was analysed as repeated measures data by fitting a mixed-effects model with Holm-Sidak post hoc test. The same tests were used to analyse the difference in the [^14^C]sucrose brain uptake between WT and 5xFAD mice. Statistical analysis of the [^3^H]amisulpride uptake (corrected for [^14^C]sucrose) into the capillary depletion analysis samples, which included the homogenate, the supernatant or the endothelial cell enriched pellet samples from WT and 5xFAD mice were analysed by unpaired Student’s t-test.

-We now state in Figure 7 legend:

Lines 908-915: ‘A: The effect of genotype in the different brain regions was analysed by Mixed-effects analysis with Holm-Sidak post hoc test. B: The effect of genotype on the capillary depletion samples was analysed by unpaired two-tailed Student’s t-test using GraphPad Prism 9. No significant effect was observed except between the supernatant samples of 5xFAD mice compared to WT mice (*P<0.05)’

-We have also added a sentence in the discussion:

Line 1198-1200: However, we could not detect any changes in [^3^H]amisulpride (corrected for [^14^C]sucrose) uptake when we compared specific brain region samples in the 5xFAD and WT mice.

**FIGURE 10**

- Due to individual variation observed in human tissue samples, we chose to analyse the different transporter samples separately using unpaired Student’s t-test to explore the effect of disease on the expression of GLUT1, P-gp or TfR1 expression.

-In the Figure 10 legend (line 965) we have now clarified that the different transporters were not necessarily assessed in the same brain sample.

**Line 981:** We now state: Details of the specific samples used in the Western blot studies are tabulated in Supplementary Table 7, S1 File.

**Q12“This suggests that antipsychotics exacerbate the cerebral hypometabolism in AD.” This is not a logical inference from the data. An impact of amisulpride on glucose uptake was not demonstrated.**

**Q12 RESPONSE:** We have ‘softened’ this statement in the abstract so that it now states: Line 53:

‘This suggests that antipsychotics could potentially exacerbate the cerebral hypometabolism in AD.’

**Q13: Despite the fact that a literature review was a highlighted part of this report, an enormous body of literature regarding glucose transporters and P-gp in AD and mouse models of amyloid accumulation was ignored in the Discussion.**

**Q13 RESPONSE:**

This manuscript cites 88 references.

The literature review method described in the manuscript was to identify groups of molecules that interacted with GLUT. It was not used to generate an exhaustive list of papers where glucose transporters and P-gp were examined in human AD and mouse models of amyloid accumulation.

However, 14 papers which describe glucose transporters and P-gp in AD are described in the discussion.

For example, references: 77, 78 and 16 focus on the 5xFAD and 3xTG mice models and wild-type controls.

For example, references 80, 81, 82, 83, 84, 19, 18, 85, 86, 87 and 88 focus on human tissue in AD and age matched controls.

**Q14 In the authors’ prior work (PMID: 31842924), amisulpride was ruled out as a P-gp substrate. Thus, it is odd that P-gp was a molecule of some intensive focus here.**

**Q14 RESPONSE:** We continue to consider that amisulpride is not a Pgp substrate. However, we were exploring the possibility that transporter expression is altered in AD and so we wanted to provide evidence that this was possible. As mentioned previously data from human tissue is rare.

**Q15 Language usage

Much of the language usage is unsophisticated or irregular.**

**Lines 44–45: “There was no significant effect of AD on mouse GLUT1 and P-gp BBB expression….” Of course, there is not; mice do not get AD. It well-recognized in the AD field that claiming (or implying) that transgenic models of amyloid accumulation are models of AD is a serious misrepresentation.**

**Q15 RESPONSE**: We have changed this sentence: Lines 44–45: **‘**There was no difference in GLUT1 or P-gp BBB expression between WT and 5XFAD mice. In contrast, caudate P-glycoprotein, but not GLUT1, expression was decreased in human AD capillaries versus controls’.

**Q16 There is a strange insertion of commas in many places that are completely uncalled for. Some of these convey an incorrect meaning to the sentences in which they appear. For instance, a comma should only separate a phrase from the item it follows if it is a nonessential phrase, i.e. one that applies to all such items. Thus, in Lines 83–84, “Amisulpride is a second-generation antipsychotic – a substituted benzamide derivative, which is a highly selective dopamine D2 receptor antagonist,” the comma indicates that all substituted benzamide derivatives are highly selective D2R antagonists. That, of course, is not true. The authors are attempting to convey that amisulpride is special among substituted benzamides in its ability to antagonize D2Rs, and that requires the absence of a comma after “derivative.”**

Q16 RESPONSE: We have removed the comma after derivative (Line 87).

**Q17 The complementary error is made in the sentence that appears in Lines 94–97. Here, comma is required after “(BBB).” Otherwise, the meaning conveyed is that there is a special subset of the BBB which controls the entry of drugs into the brain, whereas the truth of the matter is that the entire BBB contributes to this restriction. Likewise, the omission of a comma in Lines 111–112 leaves the impression that there is a class of glucose transporters that serve to induce hyperglycemia**.

**Q17 RESPONSE:** We have added a comma on line 101 and on line 127.

**Q18 In addition, use of the “Oxford comma” is inconsistent.**

**Semicolons are also used unnecessarily, as in Line 88, where a comma would be appropriate.**

**Q18 RESPONSE:** We have replaced the semicolon with a comma. Line 92

**Q19 In Lines 100–102, an incomplete sentence is inserted.**

**Q19 RESPONSE:** Thank you. We have now updated and completed the sentence (Line 106):

‘For example, differences in the expression of the multi-drug and toxin extrusion protein 1 (MATE1; SLC47A1)) protein and the plasma membrane monoamine transporter (PMAT; SLC29A4) protein in brain capillaries isolated from healthy individuals compared to AD patients have been observed (16).

**Q20 Line 211: “plastic wear” should be “plasticware.”**

**Q20 RESPONSE:** Thank you. We have corrected this. (now Line 235).

**Q21 What does the “self” in “self-inhibition studies” mean? Glucose? If it is unlabeled glucose, it is not “self” with respect to tritiated glucose. This is strange terminology for a cold-competition experiment.**

**Q21 RESPONSE:**

This terminology allows separation of two types of cold competition experiments so self-inhibition experiments (i.e. radiolabelled glucose and unlabelled glucose) from cross-inhibition experiments (i.e. radiolabelled glucose and unlabelled substrates and inhibitors). Self-inhibition experiments allow determination of transporter saturability and the measurement of kinetic constants (K_m_ and V_max_) if appropriate. Cross-competition experiments allow identification of the transporter, determination of transporter sensitivity to drugs, determination of transporter ion dependence and determination of transporter inhibition constants (K_i_) as required.

We have now added a sentence to explain this in more detail. (Line 351).

**Q22 Lines 276–277: The following is odd: “The 4 mM total concentration of non-labelled D-glucose included the standard 1.1mM non-labelled D-glucose normally present in accumulation buffer.” Are the authors attempting to convey that they did not add an additional 4 mM glucose on top of glucose that pre-existed in the buffer at 1.1 mM? Why would anyone assume that they did? The meaning of “total” should be clear to any reader**.

**Q22 RESPONSE:** We think it is open to misinterpretation, so we prefer to include this sentence to avoid doubt. We have, however, refined the explanation on line 320. See response to Q23.

**Q23 The sentence that follows that one is also odd: “Radioactivity uptake was compared to the control condition in which the cells were treated with accumulation buffer of the same composition apart from the non-labelled D-glucose content which was 1.1 mM (the standard non-labelled D-glucose concentration in the accumulation buffer).” Is this to say that the control was exactly the same as the experimental treatments except that the concentration of non-labelled glucose was lower by 1.1 mM (i.e., totaled 2.9 mM)? If they are attempting to convey that the control condition was identical except that the concentration of non-labelled glucose was 1.1 mM (i.e., lowered by 2.9 mM), a comma would be needed after “content.” Regardless, it is not clear how this is a control. Is it** **meant to be a positive control under the assumption that more radiolabeled glucose would be taken up when the unlabeled competitor was at a lower concentration? This is a very strange way to describe a cold-competition experiment. Moreover, a 3.6-fold elevation of cold competitor is hardly enough to achieve a blockade of uptake.**

**Q23 RESPONSE**: We have now modified this sentence.

It is now as follows: Line 320:

‘Radioactivity uptake was compared to the control condition in which the cells were treated with accumulation buffer of the same composition apart from the additional 2.9mM non-labelled D-glucose of the test condition’.

It is a baseline control. We would like to highlight that we have referred to preliminary experiments where we explored the use of higher concentrations of glucose i.e. 4, 6 and 9 mM in the method (lines 330-332) we state:

‘The non-labelled D-glucose concentration for the treatment condition (4 mM) was selected based on previous reports that normal plasma glucose concentrations are kept in a narrow range between 4 and 8 mM in healthy subjects (41) and on preliminary experiments studying the effect of 4, 6, and 9 mM non-labelled D-glucose on the membrane integrity of hCMEC/D3 cells (see Supplementary Fig 2, S1 File)’.

In the supplementary file on page 18 we explain:

‘There was no statistically significant effect of non-labelled glucose at a total concentration of 4 mM, 6 mM or 9 mM glucose on cell membrane integrity as measured by [^3^H]mannitol. However, individual wells in the 9 mM non-labelled glucose had very high V_d_s, suggesting decreased cell membrane integrity in those wells. As a result, 4 mM non-labelled glucose was selected to test saturable transport of glucose in subsequent accumulation assays’.

We have also taken this opportunity to remove one of the two Supplementary figures in figure 2 as it is preliminary data and the complete data set is illustrated in Figure 5A of the main manuscript. We have updated the text in the supplementary file accordingly.

**Q24 The formatting of the manuscript was very unusual**. **While the figures were compiled at the end, figure legends were interspersed throughout the main body of the text. This made the paper rather difficult to read/evaluate.**

**Q24 RESPONSE:** This type of formatting is requested by the journal.

**Q25 Reviewer #3: The manuscript titled "Interaction of amisulpride with GLUT1 at the blood-brain barrier. Relevance to
Alzheimer’s disease" by Boyanova and colleagues investigated the possible interactions of amisulpride (an antipsychotic). Overall, it is a very good manuscript but has some short-term. GLUT1 and GAPDH on the same blot at such a low resolution, but also the absence of GLUT1** **band in HEK293 cells. HEK293 cells express a non-negligible amount of GLUT1, which suspects that the antibody is not suited or not good enough. I recommend to perform this blot with SA0377, a Rb monoclonal GLUT1 antibody targeting the C-terminal fraction of the transporter.**

**Q25 RESPONSE:** The GLUT1 Western blots are found in Supplementary Figure 6 and Supplementary Figure 12.  In supplementary figure 6 tubulin was used as a loading control. In supplementary figure 12 GAPDH was used as a loading control.  Importantly HEK 293 was as specified in the legend used as a negative control.  We agree, as the reviewer states, it does not express GLUT1.  We did trial a different GLUT1 antibody, but this required a high amount of protein per well.  So we selected #ab115730 for further studies.  We have now updated Supplementary Fig 12 with higher resolution blots.  We have also taken the opportunity to add the tubulin antibody information to the Supplementary Table 1.

**Q26 Minor issue: Please show the data as Mean+SD, also provide the P-value if P<0.10 so the reader can assess is there is a trend or not to significance, and if the authors have the Km (substrate) and IC50 (inhibitor) for GLUT1 of the compounds listed. These can help the reader appreciated the selectivity and affinity of these compounds to GLUT1.**

**Q26 RESPONSE:**

26a) We have presented the data as dot plots and bar graphs reporting the mean ±SEM (Fig 2, Fig 4, Fig 5, Fig 7, Fig 10, Fig S2, Fig S3, Fig S9, Fig S10). The dot plots allow us to observe the variability of the data to the mean (similar to the standard deviation) and the standard error allows us to measure how far the sample mean is likely to be from the true population mean. We have also reported median values where appropriate (Table 1). So as we report the data as dot plots as well as bar graphs we prefer to present the data as mean ±SEM

26b) We had included the IC_50_ for the antipsychotics that interact with GLUT (Table S6). However, we now specifically mention this in the results text Line 827:

‘Literature review confirmed that other anti-psychotics inhibit glucose uptake at these concentrations. The half-maximal inhibitory concentration (IC_50_) of these glucose-transport inhibiting antipsychotics ranging from 2-100 μM (Supplementary Table 6, S1 File).’

We now also specifically mention this in the discussion text Line 1130:

‘Although it is noted that other anti-psychotics drugs have been shown to inhibit glucose uptake at these test concentrations (2-100μM) (Supplementary Table 6, S1 File)’.

26c) We have reported different levels of signicance throughout the manuscript. However, we had only placed this information in the text. We have now repeated this information in the figure legends where appropriate e.g. Fig 2 legend, Fig 4C legend, Supplementary Figure 3.
