# Supplementary material for: Interaction of amisulpride with GLUT1 at the blood-brain barrier. Relevance to Alzheimer’s disease
Source: PLoS One. 2023 Oct 24;18(10):e0286278. doi: 10.1371/journal.pone.0286278 (PMC10597500; doi:10.1371/journal.pone.0286278)
Supplement: Attachment — Submitted filename: Response to Reviewersv2.docx [file pone.0286278.s004.docx]

**COMMENTS FROM EDITORIAL TEAM:**

Please review your reference list to ensure that it is complete and correct. If you have cited papers that have been retracted, please include the rationale for doing so in the manuscript text, or remove these references and replace them with relevant current references. Any changes to the reference list should be mentioned in the rebuttal letter that accompanies your revised manuscript. If you need to cite a retracted article, indicate the article’s retracted status in the References list and also include a citation and full reference for the retraction notice.

**Additional Editor Comments:**
Two reviewers have accepted the manuscript and revision 1, respectively. Reviewer 3 made further comments on revision 1. The selection of the methods and models to study glucose uptake in culture and in mice is scientifically justified. I suggest the authors to cite two further papers in which *in situ* brain perfusion is used and brain glucose uptake is given as brain distribution volume (Pan et al. 2022 PMID: 36559296; Shah et al. 2015 PMID: 25925411). Please correct the incomplete sentence and remove the duplicate reference.

**RESPONSE FROM AUTHORS:**

1. We would firstly like to thank the editor and editorial team for their time spent reviewing the manuscript.
2. We would like to mention that we measured amisulpride and sucrose, *not glucose*, uptake using the *in situ* brain perfusion method.
3. We have now added two papers to justify the *in situ* brain perfusion method. These are papers which use the same method, are from our group at King’s College London and one also examines the test molecule, amisulpride, but in a different animal model of AD. The papers are:
a) Sekhar, G.N., Fleckney, A.L., Boyanova, S.T., Rupawala, H., Lo, R., Wang, H., Farag, DB, Rahman, KM, Broadstock, M., Reeves S. and Thomas, S.A. Region-specific blood–brain barrier transporter changes leads to increased sensitivity to amisulpride in Alzheimer’s disease. Fluids Barriers CNS 16, 38 (2019). <https://doi.org/10.1186/s12987-019-0158-1>.
b) Sanderson, L., Khan A. and Thomas, S.A. Distribution of suramin, an antitrypanosmal drug, across the blood-brain and blood-CSF interfaces in wild-type and P-glycoprotein transporter-deficient mice. Antimicrobial Agents and Chemotherapy 51: 3136-3146 (2007). <https://doi.org/10.1128/aac.00372-07>
4. We have corrected the sentence and removed the duplicate reference.
5. We have also further proofread the manuscript and added missing abbreviations (TBS-T, GAPDH), corrected and moved an ethics statement (page 10, line 233), clarified further some methological details (page 24, line 619-622). Specified that the antipsychotics were GLUT interacting where appropriate (so not simply stated antipsychotics e.g. Table 1). We have also correctly placed the endothelial cell label in Fig 8a.

**Reviewer #2:** This manuscript has been revised by making minor changes to the text and some of the supplemental figures. The most significant problems with the manuscript are:
1. The unusual and unverified “in situ perfusion” assay of brain glucose uptake. This is an indefensible assay for CNS glucose uptake and utilization.

**RESPONSE FROM AUTHORS:**

We would firstly like to thank the reviewer for their time spent reviewing the manuscript.

We did not measure **glucose** uptake using the *in situ* brain perfusion method in this study.

We assessed amisulpride and sucrose uptake.

We have now added two method references to the paper (as described above). These papers justify and verify the use of the method used in the submitted manuscript. As mentioned by the editor this method has also previously been used by other research groups (Pan et al. 2022 PMID: 36559296; Shah et al. 2015 PMID: 25925411).

2. **Reviewer #2:**  The inability of the authors to detect and quantify GLUT1 by western blot.

**RESPONSE FROM AUTHORS:**

We have been able to detect GLUT1 protein expression. We comment on this in more detail in response to question 14 from reviewer #2.

**3. Reviewer #2** The use of the hCMEC/D3 cell line to model glucose uptake by cerebral endothelial cells. Transformed cells have dramatically different glucose utilization, and this likely explains why assays with this line did not support the hypothesis inspired by *in silico* approaches and prior evidence.

**RESPONSE FROM AUTHORS:**

Although we could not find a published paper that examined glucose utilization in the hCMEC/D3 cells we have now further explained the limits of the assay in the paper. We have mentioned:

**Page 48, line 1241:**

‘However, these results are difficult to interpret conclusively as the immortalized cell line may not utilize or transport glucose in the same manner as cells *in vivo*’.

**4. Reviewer #2** Together, these issues disqualify the study.

**RESPONSE FROM AUTHORS**:

We disagree that the study should be disqualified.

Reviewer #2 is incorrect, please see responses above and those from the editor and the other reviewers.

**5. Reviewer #2** The author’s wrote an extensive response to the reviewers. Some of the authors’ responses are provided below in quotation marks, followed by this reviewer’s responses introduced by asterisks:

“Firstly, we are not saying that amisulpride may alter glucose uptake by GLUT1 and that this applies to other antipsychotic drugs.”

* From the Discussion: “Our *in silico* molecular docking studies revealed that amisulpride interacts with GLUT1 and could potentially affect glucose delivery to the CNS. …The implications of our findings extend to other antipsychotic drugs.”

**RESPONSE FROM AUTHORS**:

The statements above have been taken out of context and the original question and the full explanations provided in the first response to reviewers comments are not being considered in their entirety.

**The original query was:**

**Q3 revision one comments . Reviewer #2: This manuscript reports a gravely flawed analysis of the interactions of an antipsychotic drug (amisulpride) with aspects of cerebrovascular transport, including attempts at relevance to Alzheimer’s disease. The approach included literature review to identify and characterize confirmed and proposed antipsychotics that interact with glucose transporter 1 (GLUT1), in-silico modeling of amisulpride with GLUT1, in-vitro tests of glucose uptake in a transformed endothelial cell line, and a in-vivo perfusion assay of amisulpride uptake into the brain of a mouse model of amyloid accumulation. Levels of some transport enzymes (including GLUT1) in mouse and human brain were also assayed, and the ultrastructural integrity of cerebrovascular structure was evaluated in an Alzheimer’s case. Despite the lack of physicochemical data to this effect, the authors conclude that amisulpride may alter glucose uptake by GLUT1 and that this necessarily applies to other antipsychotic drugs. They also conclude that levels of GLUT1 are not altered in AD or a mouse model of amyloid accumulation and that P-gp levels are lower in AD brains but only in the caudate.**

Our full response to this was as follows:

Firstly, we are not saying that amisulpride may alter glucose uptake by GLUT1 and that this applies to other antipsychotic drugs.

We have *in silico* evidence that amisulpride interacts with GLUT1 and other groups have evidence that other antipsychotic drugs interact with glucose transporters including GLUT1. In fact, 11 antipsychotics were identified which interact with glucose transporters (see Supplementary Table 5, S1 File).

So there is evidence from other sources that would suggest amisulpride has potential to interact with GLUT.

Secondly, we also did not assess ‘enzymes’ we assessed the expression of ‘transporters and receptors’.

Thirdly, the physicochemical characterization data does not allow us to rule out that amisulpride interacts with GLUT.

Regarding the physicochemical characteristics:

-The +1 charge of amisulpride is similar to the other antipsychotics that interact with the glucose transporters.

-In addition, we identified one GLUT1 substrate that has a +1 charge so there is evidence that other types of molecules that interact with GLUT 1 have a similar charge.

-The MW of amisulpride suggested it would more likely be an inhibitor of GLUT1 transport than a GLUT1 substrate due to its similarity to other GLUT1 inhibitors.

**6. Reviewer #2**
“Secondly, we also did not assess ‘enzymes’ we assessed the expression of ‘transporters and receptors’.”

* Transporters (and many receptors) are indeed enzymes. If all 14 authors fail understand this, it is a shocking and troubling commentary on biochemical literacy among researchers.

**RESPONSE FROM AUTHORS:**

Transporters, receptors and enzymes have different gene names. Transporters are not enzymes.

Transporters carry substrates across a membrane.

Enzymes catalyse reactions and will alter the structure of a substrate.

**7. Reviewer #2**

“Thirdly, the physicochemical characterization data does not allow us to rule out that
amisulpride interacts with GLUT.”

* Of course it doesn’t. But scientific publishing consists of reporting what *has* been supported by evidence, not speculation about what has not. If we were to publish everything that has not been disproved, we would quickly consume the bandwidth of the entire internet.

**RESPONSE FROM AUTHORS:**

We have described *in silico* evidence, physicochemical evidence, and literature review evidence, which suggests that amisulpride can interact with GLUT1.

**8. Reviewer #2**

“However, the plasma used in the perfusion assay is artificial and as a result the concentration of glucose in the plasma is stable throughout the experiment.”

* The problem with anesthetics is not with the effects of somatic physiology on blood glucose levels; the problem is that these drugs alter the function of glucose transporters and other mechanisms directly involved in glucose utilization in the CNS.

**RESPONSE FROM AUTHORS:**

As mentioned earlier in response to question 1 (reviewer#2) we do not examine glucose transport or glucose utilization using the *in situ* brain perfusion method.

However, both the control and test results were from animals that were anaesthetized using the same anaesthetic /route of administration so the comparisons and conclusions are appropriate.

9. **Reviewer #2**

“On line 450 *(now 469)* we state that the: ‘The mouse brain was *in situ* perfused via the heart with artificial plasma.’”

* That line appears under the subsection titled “Western blot studies.” (Moreover, “heart” is rather vague. Perfusion via the left ventricle would be quite different from perfusion via other chambers.) The subsection titled “In situ brain perfusions” made no mention of the perfusion route in the original document, so it seems that this was a fair criticism.

**RESPONSE FROM AUTHORS:** We did alter the manuscript and we did thank the reviewers for their constructive comments so we have already agreed that this point was valid.

We have now more clearly specified the route of perfusion in the Western blot studies section and we have also now mentioned that the method is described in more detail elsewhere in the document.

**Page 19, line 470:** Specifically, we state: ‘The mouse brain was *in situ* perfused via the left ventricle of the heart with artificial plasma containing radiolabelled amisulpride and radiolabelled sucrose for 10 minutes as described below.’

10. **Reviewer #2**
“We would like to highlight the following paragraph to the reviewers [regarding the suitability of the hCMEC/D3 cell line for glucose uptake assays]:

* The authors provide some information about the various glucose transporters expressed by this cell line and conclude “there is consensus that GLUT1 is the most highly expressed transporter in the hCMEC/D3 cells and the BBB.” The existence of GLUT1 in hCMEC/D3 cells was never disputed. But this alone does not make the cell line sufficiently similar to cerebral endothelial cells. The poor relevance of hCMEC/D3 cells may explain the fact that the authors “could not detect any interaction of amisulpride with GLUT1 in this assay.” But the authors would rather defend their poor experimental design than develop a different one that would defensibly test their in silico predictions. Moreover, the authors failed to address this reviewer’s chief concern, namely that oncogenic transformation with viral large T antigen dramatically alters glucose metabolism. The authors might be advised to research information regarding the Warburg shift.

**RESPONSE FROM AUTHORS:** Please see response to reviewer #2 question number: 3 (second set of comments).

11. **Reviewer #2**
“We have now more clearly explained the expression of results section [regarding the report of glucose uptake in units of volume]. …In this way we can explore the means of cell entry and even cell exit of the test molecules. Movement across the cell membranes may be by passive diffusion, transporter mediated or indeed involve vesicles which may be receptor mediated.”

* The authors go to great pains to explain a frankly inane computation that relates the amount of radiolabeled glucose found in the cellular compartment to the volume that it originally would have occupied whilst diluted at its original concentration in the assay medium. It seems that the authors need to have explained for them the theory that transporters (e.g., GLUT1) specifically arrest one single molecule of glucose at a time and move it, independent of the aqueous solution in which it is dissolved, from extracellular fluid to cytosol. This has no relationship to volume whatsoever. To report the results in terms of volume suggests that the cell is drinking a liquid that contains a constant concentration of glucose. Indeed, the authors suggest this with their mention of vesicular transport. In 39 years of intense study of cell biology, this reviewer has never encountered evidence that cells emit vesicles to scoop up their liquid surroundings and then transport those vesicles back into their interior; this would be an entirely novel means of import. Regardless, it would also be quite irrelevant to the function of GLUT1, which is what these experiments are intended to address.

**RESPONSE FROM AUTHORS:**

The accumulation assay results are expressed as a volume of distribution (ml.g^-1^ of protein). This is an accepted and standardized expression of the results/data.

We do not measure the transport of one molecule by one transporter.

In this assay the concentration of the test molecule is measured and used to calculate a volume of distribution.

We do not specifically state that vesicles are released into the exterior. Although the contents of vesicles can be released into the exterior.

12. **Reviewer #2**

“We have replaced the supplementary Figure 4 images with higher resolution images. In these images you can see the APP in the 3xTG lane.”

* Image resolution was not the problem. The problem was that there was an apparent discrepancy between nominal genotype and the appearance of APP. The authors reply that the issue was related to sloppiness, not something one likes to hear from a scientist.

**RESPONSE FROM AUTHORS:**

The authors have not used the word ‘sloppiness’. An incorrect figure was uploaded to the supplementary file. This has now been corrected.

13. **Reviewer #2**

“The antibody manufacturer advises against boiling the samples…. Yet due to sensitivity issues with a previous GLUT1 antibody used, we had tested in the lab, heating (95°C or 100°C), and not heating (room temperature), and the heating condition was picked due to the stronger signal obtained.”

* The fact remains that the blots in Suppl. Fig. 6 show that GLUT1 was not effectively detected by these methods and therefore could not have been accurately quantified. This was also noted by Reviewer 3. It is impossible to surmise what “band” the authors may have tried to quantify for Fig. 10.

**RESPONSE FROM AUTHORS:**

-Supplementary file 1, Figure 6: We apologise the band label had slipped. This has now been corrected.

-Figure 10 shows: **TfR1, GLUT1 and P-gp expression in human frontal cortex and caudate brain capillary lysates from human control and AD age-matched cases**. We have now ‘linked’ these graphs to the example immunoblots in the S1 file in the text. The exact figure numbers have now been included in the text page 41-42, lines 1059-1085.

**14. Reviewer #2.** The authors also misunderstood the comments from Reviewer 3 on this topic. S/he stated that GLUT1 should have been detected in HEK293 cells. Inexplicably, the authors reply, “HEK 293 was as specified in the legend used as a negative control. We agree, as the reviewer states, it does not express GLUT1.” This is refuted not only by Reviewer 3 but by Zambrano et al. (2010, PMID: 20506349), Castro et al. (2008, PMID: 18506475), and Kraft et al. (2015, PMID: 26248369), to name a few.

**RESPONSE FROM AUTHORS**

Reviewer 3 (first set of comments) stated this: ‘HEK293 cells express a non-negligible amount of GLUT1, which suspects that the antibody is not suited or not good enough.

Reviewer 2 (second set of comments) then went on to provide three papers where GLUT1 has been detected in HEK 293 cells. These were: Zambrano et al. (2010, PMID: 20506349), Castro et al. (2008, PMID: 18506475), and Kraft et al. (2015, PMID: 26248369).

a) We firstly apologise for mis-interpreting the antibody sensitivity query from reviewer 3 (first set of comments). The reviewer was, in fact, querying the use of HEK293 cells as a negative control for the expression of GLUT1 in our manuscript.
b) Reviewer 2 provided papers which suggested that HEK293 cells should, in fact, be a positive control for the expression of GLUT1.

However, two of the three papers that reviewer 2 cited, did not use the Western blot method (Table 1). They measured mRNA by RT-PCR so the reviewer has actually have not provided evidence for the presence of ‘GLUT1 proteins’ in HEK293. It is possible for GLUT1 mRNA to be present, but for GLUT1 protein not to be expressed in cells. So these two papers do not provide evidence for GLUT1 protein expression in HEK293 cells.

The third paper that reviewer 2 cited used the Western blot method, but did not specify the antibody used to detect GLUT1.

**Table 1: Publications cited by Reviewer 2.**

| **Publication** | **Protein** | **mRNA** | **HEK293** |
| --- | --- | --- | --- |
| **Kraft** et al., (2015  PMID 26248369) | Did not describe /utilize the Western blot method. | measured mRNA expression levels | HEK293 cells were acquired from the American Type Culture Collection |
| **Castro** et al. (2008, PMID: 18506475) | Did not use Western blot. | They used RT-PCR so measured mRNA | HEK293, ATCC® CRL-1573TM |
| **Zambrano** et al. (2010, PMID: 20506349) | Western blot  Did not mention antibody used. |  | The HEK293 cell line (ATCC*1* CRL-1573TM). |

c) Another point is the source of the HEK 293 cells. We used commercially prepared human embryonic kidney 293 (HEK293) cell lysates (#ab7902, Abcam, UK) as a negative control for GLUT1. In contrast all three papers in Table 1 used HEK293 cells from ATCC, USA. This may also contribute to differences in GLUT1 expression.

We have now added the source of the HEK293 cells to the manuscript (Page 7, line 150).

d) We originally selected HEK293 lysates as a GLUT1 negative control based on the data reported in Figure 1. So positive and negative controls were chosen on the basis of RNA-seq data from The Human Protein Atlas (https://www.proteinatlas.org/, last accessed: 10/10/2019).

**Figure 1:** *RNA-seq tissue/cell data for GLUT1 reported as mean pTPM (protein-coding transcripts per million), corresponding to mean values of the different individual samples from various tissues or cell lines). GLUT-1 RNA-sequencing results are reported as normalised expression (NX) values. NX value of 1 is defined as the threshold for expression of the corresponding protein (https://www.proteinatlas.org/). Green rectangular showing the positive control we used, red rectangular – showing the negative control.*

We have now further justified the use of HEK293 as a negative control for GLUT1 by adding more information about the selection of positive and negative controls in the methods section. In fact, we have now justified our selection of negative and positive controls throughout the manuscript. We have also added appropriate references. These are references 38, 39, 49, 51 and 52.

**In hCMEC/D3 cells Page 12 Line 291 now states:**

‘Positive and negative controls for transporter expression in hCMEC/D3 cells were chosen based on RNA-seq data from The Human Protein Atlas (38,39). Lysates from human colon adenocarcinoma (Caco-2) cells were used as a positive control, whilst whole cell lysate from human embryonic kidney cell line (HEK-293; (#ab7902, Abcam, UK) were used as a negative control for GLUT1 transporter expression’.

**In WT and 5XFAD mice Page 20, line 500-507 now states:**

‘Positive and negative controls for transporter expression in the mouse brain capillary lysates were chosen based on RNA-seq data from The Human Protein Atlas (38,49). They included mouse whole kidney lysates as positive controls for PMAT, MATE1 and P-gp expression. HEK293 was also used as a positive control for MATE1 and P-gp. Lysates from cultured human airway epithelial cells (Calu3) was used as a negative control for PMAT (38,50). HL-60 was used as a negative control for MATE1 and P-gp (38,51,52). The positive control for OCT1 was HL-60 and negative control was Calu3. The positive control for GLUT1 expression was Caco2 cell lysates and negative control was HEK293 cell lysates’.

**In human brain capillaries Page 26, line 559-563.**

‘Positive and negative controls for transporter expression in the human brain capillary lysates were chosen based on RNA-seq data from The Human Protein Atlas (38,49). Caco-2 cell lysates were used as a positive control, and HEK293 whole cell lysate and HL-60 were used as negative controls for GLUT1 transporter expression (38,39). HEK-293 cell lysate was used as a positive control for P-gp transporter expression (38,52).’

e) We have also now mentioned that we also used human promyelocytic leukaemia cell line (HL-60) lysate as a negative control for GLUT1 in our study. Although this is noted in the Supplementary file figure 12 and legend. We had not mentioned this additional negative control in the manuscript text. We have now added the source of these HL-60 cells Page 7, line 139 and mentioned its use as a negative control for GLUT1 expression in the human brain capillary lysates in the manuscript (Page 26, lines 583-588 and Page 42, lines 1081-1083).

15. **Reviewer #2**

“This [difference in sample sizes across different outcome measures] was due to the availability of certain processed samples in different time frames. They were not deliberately omitted it was just availability of processed samples.”

* The authors have failed to address whether there may have been biases in the conditions that caused these differences in “availability of processed samples.” The reviewer asked whether any randomization was used; evidently, there was none.

**RESPONSE FROM AUTHORS:** The samples were not randomized. We have now specifically stated this in the manuscript in Fig 10 legend (page 41, line 1067).

16. **Reviewer #2**

* “Self-inhibition” has been retained in reference to the cold-competition assay. This reviewer still feels this is non-standard (indeed, unprecedented) terminology.

**RESPONSE FROM AUTHORS:** The authors believe it is appropriate terminology. Self-inhibition terminology is used in the following publications, so it is not unprecedented.

a) Effect of Transport Inhibitors and Additional Anti-HIV Drugs on the Movement of Lamivudine (3TC) across the Guinea Pig Brain Barriers. J. E. Gibbs, T. Rashid and S. A. Thomas

Journal of Pharmacology and Experimental Therapeutics September 1, 2003, 306 (3) 1035-1041; DOI: https://doi.org/10.1124/jpet.103.053827.

b) Dogruel, M., Gibbs, J.E. and Thomas, S.A. (2003), Hydroxyurea transport across the blood–brain and blood–cerebrospinal fluid barriers of the guinea-pig. Journal of Neurochemistry, 87: 76-84. <https://doi.org/10.1046/j.1471-4159.2003.01968.x>

17. **Reviewer #2**

* The Oxford comma is still used inconsistently. This may seem like a trivial matter, but the inconsistency often requires the reader to read a sentence twice to understand the meaning.

**RESPONSE FROM AUTHORS:**

We have now reviewed each of the commas in the main manuscript and in the supplementary file and we have removed the Oxford comma.

18. **Reviewer #2**

* There is still at least one incomplete sentence (Lines 1141-1151).

**RESPONSE FROM AUTHORS:**

We have now revised these sentences (now lines 1248-1261).

17. **Reviewer #2**

* References 23 and 27 duplicate one another.

**RESPONSE FROM AUTHORS:**

We have removed the duplication of reference 23.
